# Relapse Experience in Iranian Opiate Users: a Qualitative Study

**Published:** 2014-04

**Authors:** Naiemeh Seyedfatemi, Hamid Peyrovi, Amir Jalali

**Affiliations:** 1 Center for Nursing Care Research, Iran University of Medical Sciences, Tehran, Iran;; 2 Department of Psychiatric Nursing, Kermanshah University of Medical Sciences, Kermanshah, Iran

**Keywords:** Relapse, Drug Users, Psychosocial Experience, Qualitative Study

## Abstract

**Background:** To understand the relapse process, it is required to notice the clients learned behaviors and environmental contexts. We aimed to explore and describe relapse experiences of Iranian drug users.

**Methods:** This is a grounded theory study and twenty two participants were selected using purposive sampling, snowball and theoretical sampling. After obtaining written informed consent, data gathering was done by means of in-depth semi-structured interviews. According to Strauss and Corbin three phases of open coding, axial coding and selection coding were done for qualitative analysis and continuous comparison. During the research period Guba and Lincoln criteria were used to be reassured of the accuracy and rigor of the study findings.

**Results:** The main categories of this study were craving and conflict, family stress and psychological indicators of relapse that emerged in three phases including recovery, tension and pre-relapse. High anxiety, withdrawal, rationalization and lying were the most common symptoms.

**Conclusion:** Family reactions and social conditions play a key role in relapse. Relapse process is an active and multidimensional event in which the clients experience a psychosocial status continuum from recovery to relapse. Most psychological problems are seen in the tension phase.

## Introduction

Drug abuse is a common phenomenon in the world that threatens economy, families, communities, and even people’s lives. Currently, drug addiction is a great personal and social problem affecting the body and mind of users and also the health of the society, causing cultural, political, economical, and social problems.^1^ In recent years, drug abuse has become a serious and basic problem for society causing widespread social, familial, psychological, and economical hazards.^2^

Despite advances in the treatment of alcoholism and other addictions, frequent relapses and re-use of alcohol and other substances is a serious problem in the treatment of these patients.^3^ During treatment, symptoms of return to drug use often start in a short time, leading to the clients’ desire to abuse drugs.^4^^-^^6^ In various studies, the rate of relapse and return to alcohol or drugs is as high as 75percent in the first six months of therapy.^5^^,^^7^^,^^8^ Symptoms and behavioral changes in relapse and its related factors have been discussed frequently in the literature.^9^ Most of these texts focus on factors such as stress, social support, and even personality factors as predictors of relapse.^10^^,^^11^ Craving to abuse drugs is addressed in the literature as a major factor in relapse. Many variables related to psychosocial stressors and emotional status of clients such as depression, anxiety and emotional crisis are effective in treatment periods and relapse process and are key factors in relapse.^11^ Craving to use drugs is an emotional force and an obsessive thought that produces a huge force for seeking drugs and finally drug use. Craving is a factor that most drug-dependent users experience. According to DSM-IV it is a central symptom among substance disorder patients.^12^

Behavioral change is a complex process influenced by the treatment process and individual factors associated with the client.^13^^,^^14^ The most important psychological causes can be lack of confidence, cravings and emotional distress noted in the course of treatment and even aftermath that can lead to relapse.^8^ We aimed to explore the relapse experiences of substance abusers in Kermanshah city, west Iran.

## Materials and Method

This article is part of a larger project being performed using the qualitative method and the grounded theory approach. This kind of study is aimed to obtain rich data and illuminate social processes in human relations.^15^ The purpose of the study was obtaining rich data and understanding the relapse process, its components and their relationships using the experience and knowledge of people involved in relapse.

According to the purpose of the study which was understanding the relapse experience in drug abusers, 17 drug addicts having at least one relapse history and being well informed about relapse experience were chosen using purposive and snowball sampling. The first participant was selected by purposive sampling and other participants were recruited using the snowball sampling method. The study began by purposeful sampling and continued by theoretical sampling with maximal diversity. After explaining the purpose of the study to the participants and getting written informed consent, interviews were done and the conversations were tape recorded. Maximum variation regarding sex, age, job, kind of opiate, marital status was considered in sample selection. At the beginning of the interview, for warm-up, the participants were asked to introduce themselves and explain about the reasons of re-using the drugs after treatment and talk about the signs and problems of the time period between treatment to relapse and whether they had enough opportunity to figure out the events of that period. During the interview, according to the answers, the questions were focused on psychosocial outcomes in the time period between treatment and relapse.

Subsequent decisions regarding who would be interviewed were made as data collection and analysis progressed. Therefore, three family members and two physicians, as substance-withdrawal therapists, were included in this study according to theoretical sampling. A total of 25 interviews were conducted with the following participants: 17 drug users, 2 family members, and 3 physicians. Three participants were interviewed twice. All interviews were tape-recorded.

*Ethical Considerations*

The study was approved by the Ethics Committee of Tehran University of Medical Sciences. After obtaining approval, the participants were informed orally regarding the purpose of the study and signed the written informed consent form. All participants were told that they could withdraw from the study at any time they desired. They were assured of confidentiality of tape records and transcriptions. 

*Data Analysis*

Analysis of data began at the first interview. All of the interviews were done in 7 private and 3 governmental drug abuse treatment clinics in Kermanshah. All of clinics were supervised by Kermanshah University of Medical Sciences. The interviews were done in mother language by one of the researchers who had complete knowledge about the mother language of the participants. Every interview lasted for 30-70 minutes. Finally, data saturation was achieved after 25 interviews (22 participants). Data collection began in February 2009 and continued until July 2011. Data analysis and data collection began simultaneously. Corbin and Strauss continuous comparison method was used for data analysis (1998). In this method, data collection and analysis are performed simultaneously. This process began with open coding phase. In this phase interview transcripts were read carefully several times and some statements or sentences were assigned codes in the margins of the transcripts which accurately captured the respondent’s meaning. During the axial coding phase, the codes in each interview were then compared with those in each other interview to create broader categories that linked codes across the interview. The aim of categorizing was to reduce the number of codes which were similar or dissimilar into broader higher order categories. The categories were compared with one another. During comparison, some of the categories were integrated and sometimes a new class was formed inside the previous ones. This continuous comparison was done carefully until the axial coding was assigned. At the same time of data analysis, field description was done and coded in this manner. Finally selective coding defined the relationship of the classes. During the interviews and data analysis, the researcher wrote down any notes, hypotheses or relationships he found useful in research analysis. The emerging categories were analyzed by comparing and contrasting them with each other to ensure that they were exclusive and covered the variation in the data. In selective coding phase the core variable was emerged. In this article, we present part of the findings in the axial coding phase. 

*Study Rigor*

For being assured of the accuracy and rigor of data, we used the Lincoln and Guba’s evaluative criteria. During the study, the researcher (third author) had long engagement with data which reassured the participants and helped them to have appropriate understanding of the study atmosphere. Transferability was considered by having team (including authors and coauthors) consensus decisions regarding the categories. Dependability was strengthened by writing memos. Credibility was checked by long engagement and member-check procedure. The participants’ overviews (member check) was used to confirm the data and codes, which means that after each coding, the interview content was given back to every participant in order to be reassured of accuracy of codes and interpretations. Then inappropriate codes which were not showing their point of view were changed. The long term experience of one of the researchers (third author) in substance-withdrawal centers as a counselor was useful in confirming codes and interpretations. During sampling, maximum variation was considered to ensure credibility. Interviewing the family members and physicians increased data validity. Peer check and member check was done for increasing the credibility of the study. For confirming transferability, the data was given to some drug abusers who were not as the study participants to read the data. They confirmed data harmonization. 

## Results

Findings regarding demographic characteristics showed that the drug abusers (17 participants of 22) had 3 to 11 years of substance use experience. Their mean±SD age was 31.75±7.23 years (range: 23-45 years, table1).

**Table 1:** Demographic Data

**Characteristics**	**n=17**
Gender, %(n)	
Male	70.59 (12)
Female	29.41 (5)
Age (years)	
Median, range	29, 22-45
Education	
<high school	17.65 (3)
High school diploma	52.94 (9)
College or above	29.41 (5)
Marital status	
Single	52.94 (9)
Married	29.41 (5)
Divorced	17.65 (3)
Opiate use (years)	
Median, range	6.5, 3-22
The frequency of relapse	
Median, range	3, 1-4

In this study we found out psychological problems of the clients during the relapse process (since recovery to relapse) was a major factor that could affect other intervening factors for re-using drug. We found three major phases in this regard, namely quitting opiates or recovery, tension, and pre-relapse. The major categories during the quitting (recovery) phase consisted of somatic and psychological consequences. In tension phase craving and strive for being accepted and self-deficit emerged. In the pre-relapse phase, there were major indicators for returning to drugs. Moreover, two major factors including family challenges (tension and attitude) and confrontation with the same past situations throughout the process had the key roles to return drug use again.

*Recovery Phase*

During the recovery phase (a time period of two weeks after the end of drug withdrawal) the client experienced restlessness, behavioral changes and urgent need for respect as well as confronting the past similar situations and family challenges which finally led to anxiety and psychological tensions. Behavioral changes included extreme eagerness to meet addicted friends, aimless street wandering, insomnia, excessive smoking, and changes in lifestyle, diminished activity, and feelings of loneliness. One of the clients said: “After a week my body was numb, I liked to quarrel with everybody and was easily angered. I had no sense and hated everyone. I continuously imagined that I have drugs shots and felt high. After a month I felt better, but I had cravings, especially when I was alone or when I had a quarrel with my family.” One of the family members of the clients said: “Well, he was so restless in the early days and we did not let him go out. We provided everything he wanted. He was continuously feeling lonely and was angry, selfish and demanded too much. He asked for some cookies and when we bought it for him, he ate one pound at a time. His behavior with his mother was awful and he wanted different things on and on. He repeatedly said: “I have quitted” and repeated this day after day. He asked for money to go shopping, but I was afraid to give him too much. When I gave him a little, he shouted and said: it is not much and….” Another client stated that: “I think that the second week was very hard, I had no somatic pain, but drug desire was more severe. I was at home and did not go out, but I was preoccupied about the time I was using drug, its pleasure, and the way I used it and even the first time I was pleased by using heroin. So I was angry and struggled to seek excuses to be mad at everyone, get out and use drug, then blame others for that.”

The next participant said: “I had cravings, my body ached, I hated myself, I hated every body, and I was easily angry and fought every one who was speaking. In general, I felt so bad and even worse when I was angry or had cravings. I fought myself over and over to control the cravings.”

In this phase, psychological symptoms of the clients were mostly anxiety and mood and behavioral changes. But the severity of psychological problems in this step was very different.

*Tension Phase*

After ending the recovery phase, the client faced too many problems as a result of abstinence. The role of family challenges and social conditions were important. Family challenges had positive and negative (mostly negative) effects on the client’s behavior which stimulated re-use of drugs. Family and community attitude made was another challenge and affected the client’s attitude and behavior. In this phase, the category of craving and strive for being accepted was emerged. Confronting inappropriate family and society attitudes played a key role in relapse. The family’s pessimistic questions and reactions made the drug user become angry. For example, one of the clients said:”My husband and my parents did not believe that I had quitted. They were repeatedly saying that I am lying and I am still using drugs but not in front of them. Wherever I was going they followed me and did not leave me alone, even in the kitchen or bathroom they were controlling me on and on. So I was so mad at them and fought them all. Sometimes I hit myself and I was whispering that it is better to use drugs again because it is the same and they do not believe me.”

Another client stated:”Nobody helped me at home. There was not even a piece of bread at home and I had to provide everything by myself. Neighbors, they all knew me as an addict. Even when I had quitted and claimed that I’m clean and I’m not a junkie anymore, but employers never accepted to hire me. So I had to go back to committing offences to make a living. I had to sell drugs and so there was no drug use obstacle for me. I was searching help but nobody helped me. I needed monetary and psychological help. An addict is sick and needs nursing and caring, I was sick too, but nobody supported me.” 

In this period, leaving the client alone with no support by the family and community causes multiple problems for clients which affect their life. This client continued:”When I had to commit theft or prostitution, I hated myself, but when I was using drugs it was not important. When the hangover ended and I wanted to use again I hated everyone, but when I was abstinent, I tried not to do these things, but sometimes I had to. I did not have anything and for days I had nothing to do. I did these things because I had some experience, but it was so hard. I was in trouble, angry and hating myself”, then he cried.

Another problem of the clients was drug using cravings and mental challenges which made them anxious. One of the themes frequently quoted in the interviews was “coming into grips”. The clients in this phase are continuously struggling with themselves to use drugs or not. So they are always depressed, anxious and of have low tolerance. Their decision abilities were disturbed and were deeply dependent on others. One client said:”I had cravings and I hated myself, I hated everyone, I became angry easily, I was mad at every body saying something, I couldn’t sleep and I was miserable, when I became angry or felt cravings I was even worse. I kept fighting myself to control the cravings. You have no idea, when somebody quits, he likes to use drugs again but imagining past situations made me refuse drug re-use. Even when they jokingly say: let’s take something, you feel some heavy paralyzing anxiety and craving and you don’t know what to do.”

Another client said:”When quitting, all of the junkies’ bodies are sick and ready to use again and remember the previous state of providing drug. Even when being alone, they like to experience that time. They are ready to use, but because of fear of addiction situations like community rejection or fear of being left alone by family members, they fear use drugs again. When somebody uses drugs, his mind is empty and all he remembers is pleasure and he doesn’t like to lose that mood, so he uses drugs on and on. When quitting or quitted, he is ready to use, but he rationalizes that he has to use drugs and if that event hadn’t happened, he wouldn’t have used drugs…”

*Pre-Relapse Phase*

In this phase, the client is psychologically and severely disturbed. Personal challenges, especially cravings and self-deficit played the major roles in this phase. Because of severe cravings and ineffective support of the family and also placement in the same past situations, self-deficit increased and the client entered relapse markers and showed somatic and psychological symptoms. The client used rationalization frequently, his anxiety increased, his concentration declined, and he became emotionally unstable, irritable, and angered easily. He tried to be alone, preoccupied with the sweet days of drug abuse. He was not in good relations with his family members and wished to meet addicted friends. After an episode of using drugs named ”lapse”, he felt guilty, depressed and easily annoyed because of high anxiety he fought himself on and on and wandered between treatment, seeking help from family and friends or re-using drugs because of the possibility of being rejected. In this phase, the client has dual feelings: before using drugs, he experiences high anxiety, irritability, and show-off, demanding and sometimes pleading behaviors toward family. Right after using drugs, he feels highly relaxed and ignores others. His anxiety is diminished and he barely reacts to environmental stimuli. After the ecstatic phase passes, he feels guilt, self-hatred, isolation, and depression.

One of the participants said:”When somebody doesn’t use drugs for a while, his body needs drugs and when he meets old friends and some good fellows, he wishes to use it again. In general, when you quit, you like to visit your friends, even when they call you or you see their phone number on your cell phone, you feel anxious and want to visit them soon and when you encounter them, at first you say:”Oh, come on, I’ve quitted for three months.”You fight yourself in your mind, you are afraid of being addicted again and on the other hand, you can’t ignore or forget its pleasure. You like to be calm again, so you look forward to hearing from your friends offering you drugs or find some reasons to use them and then blame others for forcing you to use drugs again.” another participant stated:”I had cravings and was saying:”Oh, come on, I won’t be an addict by one episode of using and I can control its outcomes by taking some pills tomorrow.”You know, it’s too hard. When you are a just-quitted junkie, you look like an exploding bomb, you will easily be tempted and you want to get relaxed as soon as possible. In addition, there are some fears of the family and the future. I had nothing, no investment.”

Another client said:”I got one dose of opium and used it quickly. At first it was good, then I regretted it and an hour later I hated myself. I hit and punished myself for using drugs. I felt I was nobody, was cursing myself. Then I came home angry and sad. My mother was at home and told me that I have to be ashamed, slapped me in the face and told me that I should be ashamed for using drugs again. I denied it, I was so angry and shouting, but she had figured it out. I went to my room and closed the door. My father and brother came home, shouted and knocked the door many times, but I didn’t open the door.”

In this phase, the client fears being rejected by family members or even the treatment team, so he lies and denies using drugs. Many clients commit theft, prostitution or selling household objects because they don’t have enough money to buy drugs. One of the clients said:”I had some feelings like anxiety. I decided to go but I was afraid, I didn’t go that day and I came home, I was mad at everyone. I didn’t eat supper and went to bed but I couldn’t sleep. I was smoking on and on but I felt anxious, I liked to go and imagined that now they are enjoying. Eventually I took four pills of diazepam and fell asleep. Three days later, I met my friends at the neighborhood. I had called it a day and was tired, so I liked to meet them and waited for 1-2 hours to visit them. They told me again why I didn’t join them go out and take something. I refused, but I wanted to go. I kept watching them. Somebody else said:”Let’s go but you don’t use drugs.”I said:”Ok, but I won’t use drugs and I’ve quitted, but you are the looser.” 

Another client stated about the way of finding drugs:”I did everything to get drugs, committed theft or prostitution, dealt drugs or whatever I could. I was searching help but nobody helped me. I needed monetary and psychological help. An addict is sick and needs nursing, I was sick too, but nobody supported me.” Also, a client said:”The junkie always feels anxious, fights himself and his preoccupations make him search for some reasons for using drugs. When the atmosphere is ready like this, he uses easily and blames others, while he wants it himself, he wants to be calm and without anxiety. I was such a person too, but they made me be like this.”

One of the therapists stated: “The family says he is lying again, comes home late, has disturbed sleep, is bad-tempered, uses different drugs again, and uses alcohol. We notice little lapses in the tests. There are somatic signs and social problems like contact with other addicts. He has some declines in job, attends his job late, has is absent and even lies to the therapist. He swears a lot for his deeds and words and even swears the lie.” As seen, the clients experience different somatic, psychological and social outcomes during the treatment period until relapse. In the study, we achieved different somatic, familial, social, psychological, and sexual outcomes. 

## Discussion

In this study, the main purpose was to explain the relapse process in drug users which was assessed in a ground theory study. This article is part of the larger study which examines the psychological signs, problems and outcomes of the drug users in the time period of abstinence to relapse. In this study, we found that the clients experience three phases of recovery, tension and pre-relapse since abstinence to relapse. These three phases have interactions and have no clear and separate boundaries. It was clear that the clients experience psychological, behavioral, and social problems during the treatment period until relapse. The results of the study showed that psychological problems are experienced in the global category of abstinence, family challenges, personal challenges and confronting the same past situations. Different factors and themes of abstinence happened in the recovery phase. In this phase, the clients experienced irritability, behavioral changes and need to be respected, anxiety, nagging, demanding behavior and the need to be accepted were more frequent. Unpleasant emotions, conflicts and social pressure were the predisposing factors in the relapse process.^6^ Stress has a major role in relapse and the clients experienced some levels of stress during the relapse process.^16^ Wallace found that there was a personality problem like loneliness, emotional painful situations and using defensive emotional mechanisms in the clients during the relapse process.^17^

In the category of family challenges, family tensions and their inappropriate attitudes were important. The clients experienced being questioned frequently, failure in love, family quarrels, lack of confidence, and not being trusted, which all exerted much pressure on them. Elgeili and Bashir state that in the route of relapse, the clients’ experience multiple social factors which they call social pressure and believe that those are among the important reasons of relapse.^18^ Moreover, the family attitude towards the clients makes some problems in their relationship with family members and relatives. Chaney and colleagues found that interpersonal interactions such as environmental situations, negative emotional status, and direct and indirect social pressure are among the factors experienced in the relapse process and are effective in re-using the drugs.^19^ Wallace found out that in the relapse process, interpersonal interactions of the clients changed and became problematic and especially there were dual feelings in the family members toward the clients.^17^

During the tension phase, craving and concerns about being accepted were the key factors of relapse. Mental conflicts and rationalization are experienced by the clients in this phase. The client is anxious for different reasons. Self-deficit and self-management play major roles in this phase. The tolerance level of the client decreases and he constantly seeks his own comfort and imagines it all in drug using. So, clients rationalize to be relaxed and find some excuses to use drugs. Ramo found out that the clients experienced interpersonal conflicts in the relapse process.^20^

The results showed that in the pre-relapse phase, the client experiences conflicting reactions. When he uses drugs, his anxiety diminishes and he is relaxed, but later as he becomes aware of himself, he feels guilty and regretful and hates himself. Talkativeness and isolation are two different reactions of the clients. Ramo found that during the relapse process, the clients experience negative feelings and emotions and lose the ability of distinguishing social norms the self-effectiveness decreases.^20^ He also revealed that the relapse process accompanies psychological disorders in teenagers and the most important of them is depression. Wallace also found that in the relapse process, the clients show personality problems and some troubles like painful emotional situation, boredom, loneliness, depression, frustration, and anger which all affect their behavior.^17^

## Conclusion

Relapse is an active and multidimensional process in which the clients experience a psychological status spectrum from recovery to relapse. Most psychological problems are seen in the tension phase. The clients experience multiple psychological, social and behavioral problems which can affect the relapse process and lead to drug re-use or prevention. In addition, self-deficit and self-management are two basic opposing mechanisms, and by increasing cravings and personal conflicts, the mechanism deviates to self-deficit and rationalization eventually leading to re-using the drugs. We conclude that addicts show a dual emotional behavior in the pre-relapse phase and feel frustrated and need social and familial support in order to prevent relapse and help durability of recovery. 
